# Effects of Theacrine as a Pre-Workout Supplement

**DOI:** 10.3390/ijerph192114037

**Published:** 2022-10-28

**Authors:** Henrique Santa Capita Cerqueira, Hugo Tourinho Filho, Marcos Corrêa Junior, Carlos Eduardo Martinelli Junior

**Affiliations:** 1 Ribeirao Preto Medical School, University of Sao Paulo, Ribeirao Preto 14876-018, Brazil; 2 School of Physical Education and Sport of Ribeirao Preto, University of Sao Paulo, Ribeirao Preto 14040-900, Brazil

**Keywords:** physical education and training, dietary supplementation, alkaloids

## Abstract

The search to increase physical performance is inherent to physical activity practitioners, and nutrition features are among the alternatives to seeking such an increase. The literature from the area has shown that different substances can promote beneficial effects over physical performance. One substance that has come into the spotlight is theacrine, an alkaloid similar to caffeine, which aims to increase physical performance. However, the studies on this supplement are scarce. Therefore, this study is a randomized, controlled trial that aimed to verify the effects of theacrine supplementation over physical performance in young male athletes, by applying a battery of physical tests. Twenty-two male amateur flag-football athletes were recruited. Subjects were divided into two groups and assessed at two moments, which were 72 h apart. The first assessment served as a basal measurement. In the second, the subjects ingested the supplement or a placebo 60 min before the following tests: sextuple jump, agility T test, 30 m sprint, 40 s run test (Matsudo test), and 12 min run test (Cooper test). There was no difference between the groups in any of the tests. Therefore, the findings of this study do not support the use of theacrine to increase physical performance.

## 1. Introduction

Most athletes use dietary supplements with the aim of increasing their performance [1]. The use of pre-workout supplements to increase physical performance has increased dramatically in recent years [2,3]. Caffeine is one of the most-used supplements for this purpose in the world, which has stimulating effects that are well established in the literature and promotes an increase in performance in different types of modalities [4]. However, caffeine is also known to cause various side effects, such as tachycardia, agitation, insomnia, etc. [4,5]. The substance was even prohibited by the World Anti-Doping Agency (WADA); it only became permitted in 2004 and, even so, it is still included in the category of substances monitored by the agency [4,6].

In addition to the aforementioned side effects, there is concern that people can develop caffeine dependency; there is, therefore, an interest in seeking alternatives to consuming this supplement [7]. One alternative that has come into the spotlight is theacrine, an alkaloid with a similar structure to caffeine that is used with the aim of providing the same effects, but without the side effects [8,9]. Theacrine is mainly found in Cammelia Assamica var. Kucha (Kucha) and has a similar structure to caffeine, including its probable mechanism of action, acting as an antagonist of adenosine receptors [9,10]. Theacrine is widely marketed as a pre-workout supplement, with claimed benefits of improving physical performance, but without side effects such as tachycardia, agitation, tremors, which is common with caffeine use. Furthermore, it is claimed that theacrine would not undergo adaptation/habituation, as caffeine does [11,12].

Despite its major commercial appeal, the literature on the topic is quite scarce, with most studies having assessed the safety of using theacrine or its benefits to health in general [9]. To our knowledge, only two studies in the literature have acutely evaluated the effects of theacrine on physical performance, with one assessing the effects of supplementation on strength and the other the effects on aerobic performance [13,14], in addition to another study that evaluated the chronic effects of theacrine on physical performance [15].

Therefore, the aim of this study was to evaluate the effects of acute theacrine supplementation, not only on strength and aerobic performance, but also on agility, sprint capacity, and anaerobic performance.

## 2. Materials and Methods

### 2.1. Participants

**Figure 1:**
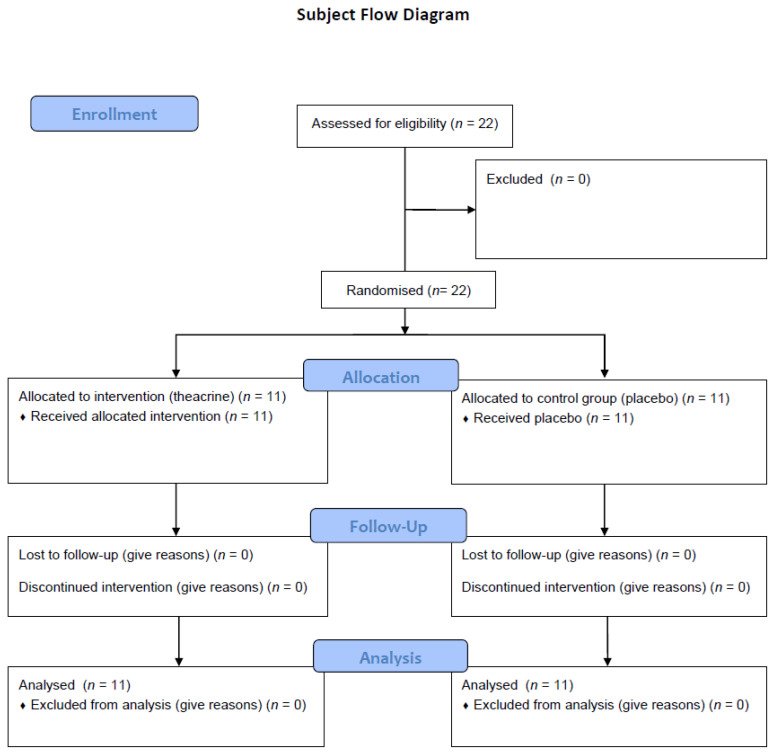
Subject flow diagram.

We recruited 22 male amateur athletes aged between 19 and 24 who practiced flag football. Convenience sampling was used according the acceptance and availability of the athletes to take part in this study. The inclusion criteria were defined as: being between 18 and 25 years old and having at least one year’s experience in the modality. The exclusion criteria were using any other type of supplementation and using or having used any type of anabolic steroid. All the subjects received and signed a free and informed consent form, in which they were informed of the entire configuration of the study, and possible risks. The article followed all the ethical issues required for research related to exercise science [16]. The study was approved by the Research Ethics Committee of the Clinical Hospital of the Ribeirão Preto Medical School–HCFMRP (USP) (CAAE: 03107618.3.0000.5440; decision n. 3.089.224). Figure 1 shows subject flow diagram, according to the CONSORT 2010 statement.

### 2.2. Protocol

This study is a randomized, double-blind clinical trial. The participants were assessed at two moments 72 h apart. In the first evaluation, used as a control, subjects carried out the physical tests without ingesting any type of supplementation. In the second assessment, 3 days later, the subjects ingested a capsule containing theacrine or a placebo 60 min before repeating the tests.

The tests used were: sextuple jump [17], agility T test [18], 30 m sprint [19], 40 s run test (Matsudo test) [20] and 12 min run test (Cooper test) [21]. An anthropometric assessment of the subjects was also carried out before the first battery of tests. Height, body mass, fat mass and lean mass were determined. For this, a stadiometer (AVA-312, Avanutri, Três Rios, Brazil) and bioimpedance scale (BF 1000, Beurer, Ulm, Germany) were used.

### 2.3. Statistical Treatment

A mixed effects linear model was used, and a 0.05 significance level was adopted. The variables are expressed in median and interquartile interval or, when appropriate or indicated, in mean and standard deviation. After a detailed description of the data, mixed-effects linear models were adjusted to compare the means of the groups and moments of interest. The multiple comparisons were carried out through orthogonal contrasts estimation, obtaining the differences between the means and their respective 95% confidence intervals. For the analyses, PROC MIXED from the SAS 9.4 software (SAS Institute Inc., Cary, NC， USA) was used.

The subjects were randomized and divided into two groups using Random Allocation Software [22]. One of the groups received capsules with 200 mg of theacrine (T group), while the other group received placebo capsules, which were equal in weight and size (P group). On the day of the second battery of tests, a member of the team distributed the capsules, which the subjects ingested 60 min before the tests.

## 3. Results

Results are expressed in Table 1 and Table 2 and in Figures. The values are expressed in mean ± standard deviation (SD).

Table 1 shows the general characteristics of the subjects. As shown in the table, there was no significant difference between the subjects from both groups in any of the anthropometric parameters evaluated. Table 2 shows the performance of the subjects in the first and second batteries of tests. No significant different was found between the groups for any of the tests.

### 3.1. Sextuple Jump

**Figure 2:**
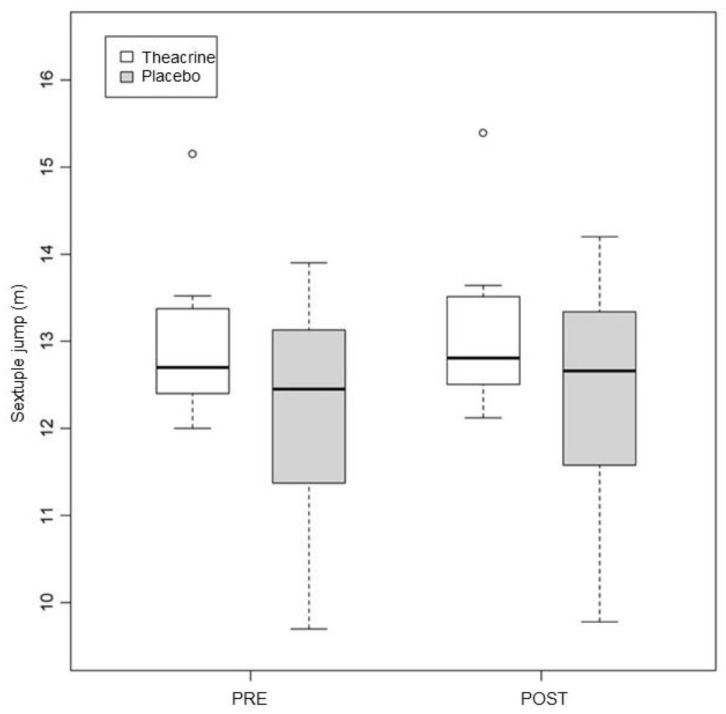
Alterations in performance in the sextuple jump in the first and second assessments. Values expressed in median–interquartile interval. m = meters.

The pre vs. post values were 12.96 ± 0.89 vs. 13.09 ± 0.92 in the T group (*p* = 0.03) and 12.18 ± 1.31 vs. 13.36 ± 1.37 in the P group (*p* < 0.01) (Figure 2). No differences were found between the groups (*p* = 0.90).

### 3.2. Agility T Test

**Figure 3:**
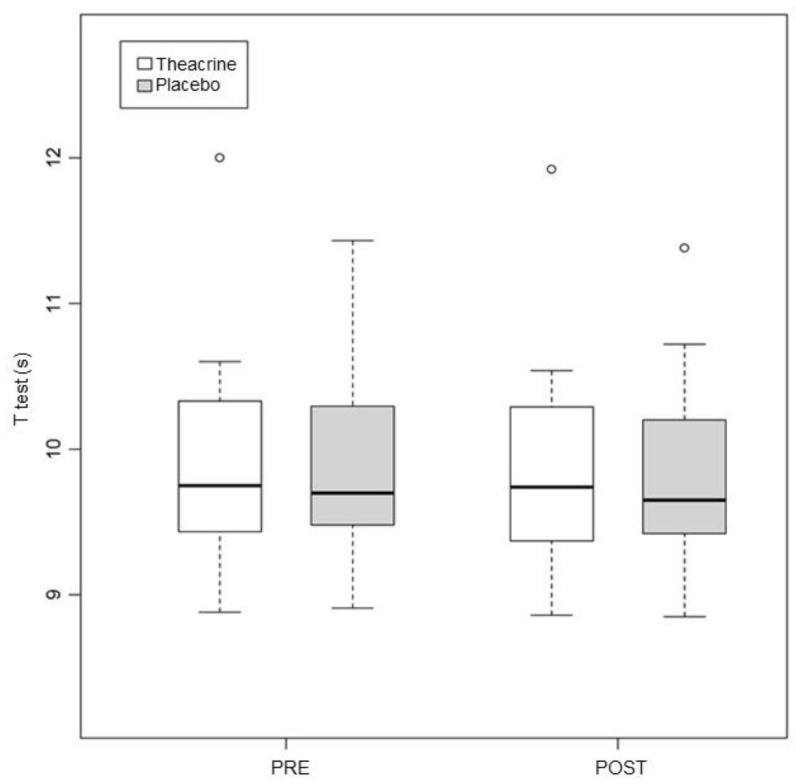
Alterations in performance in the T test in the first and second assessments. Values expressed in median–interquartile interval. s = seconds.

No intra or intergroup differences were found. T group: 9.96 ± 0.86 vs. 9.91 ± 0.85 (*p* = 0.42); P group: 9.94 ± 0.73 vs. 9.87 ± 0.73 (*p* = 0.20) (Figure 3). The *p* value in the comparison between the groups was 0.90.

### 3.3. 30 Meter Sprint

**Figure 4:**
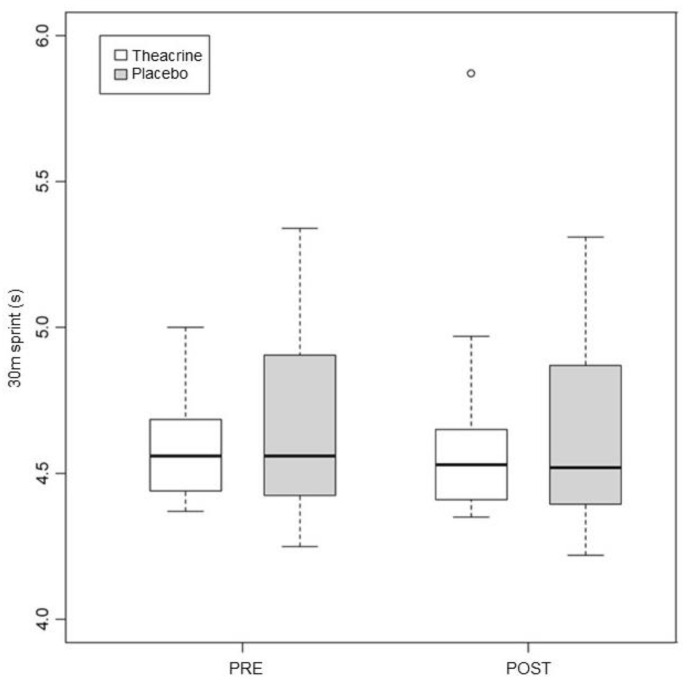
Alterations in performance in the 30 m sprint in the first and second assessments. Values expressed in median–interquartile interval. s = seconds.

The pre vs. post values in the T group were 4.69 ± 0.44 and 4.66 ± 0.44, respectively (*p* = 0.01); in the P group, they were 4.69 ± 0.36 and 4.65 ± 0.35 (*p* = 0.01) (Figure 4). The *p* value in the comparison between the groups was 0.95.

### 3.4. 40 Second Run

**Figure 5:**
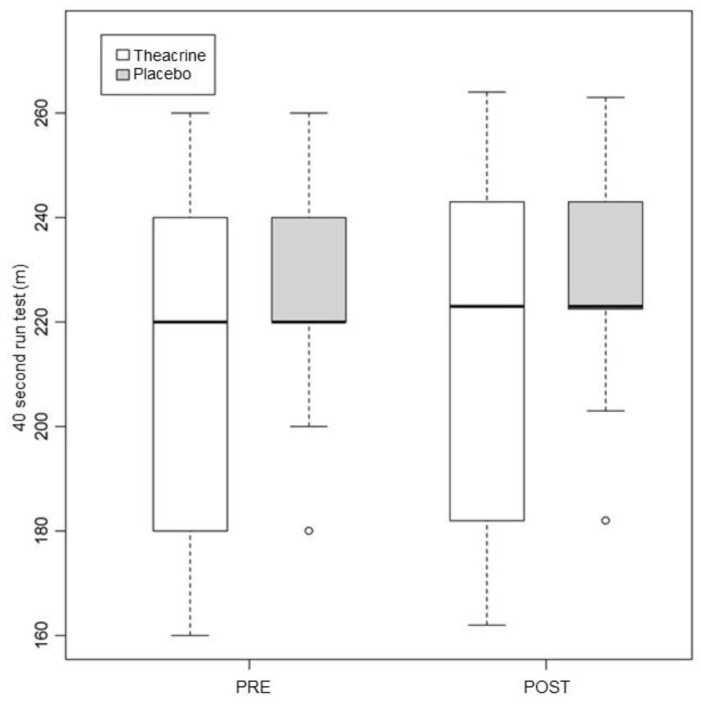
Alterations in performance in the 40 s run in the first and second assessments. Values expressed in median–interquartile interval. m = meters.

In this test, the pre vs. post values in the T group were 212.73 ± 34.95 and 215.36 ± 35.85, respectively (*p* = 0.01); in the P group, they were 225.45 ± 22.07 and 228.27 ± 22.31 (*p* < 0.01) (Figure 5). The *p* value in the comparison between the groups was 0.32.

### 3.5. Cooper Test

**Figure 6:**
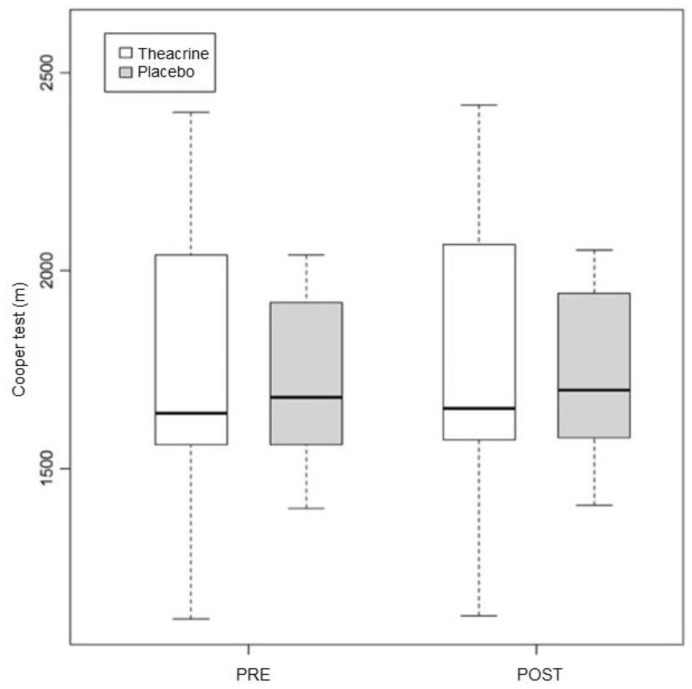
Alterations in performance in the Cooper test in the first and second assessments. Values expressed in median–interquartile interval. M = meters.

In this test, the pre vs. post values in the T group were 1745.45 ± 391.21 and 1761.09 ± 395.63, respectively (*p* = 0.02); in the P group, they were 1734.55 ± 221.47 and 1749.82 ± 223.51 (*p* = 0.02) (Figure 6). The *p* value in the comparison between the groups was 0.94.

## 4. Discussion

This study aimed to assess the effects of theacrine on physical performance in an acute way, in a battery of tests. For this, tests were employed to measure strength, agility, sprint capacity, as well as aerobic and anaerobic performance. Despite the indications for using theacrine, in our study, we did not find any positive effect of the supplement on the athletes’ performance. Previous studies also have not found any benefits of theacrine on physical performance [13,14].

One of those studies, conducted by Bello et al. (2019), evaluated 24 footballers of both sexes (10 men and 14 women) [13]. The subjects carried out the tests in four different conditions, namely: 275 mg of theacrine (TCr); or 275 mg of caffeine (Caf); or both combined, with 125 mg of theacrine and 150 mg of caffeine (TCr + Caf); or ingesting a capsule containing a placebo substance. A treadmill run test was conducted until fatigue, at an intensity of 85% VO2max, as well as cognitive tests. The run test was conducted after a football match simulated on a treadmill (the athletes ran for 45 min, rested for 15 min, then ran for another 45 min, at similar intensities to those executed during a typical football match). The cognitive tests were conducted during the interval and straight after the second period of the simulated match. There was no significant difference between any of the groups for all the evaluated parameters. The authors found only a tendency for improvement for the three intervention groups when compared to the placebo group.

The other study, from Cesareo et al. (2019), assessed 12 men trained in a crossover model, which evaluated the acute effects of theacrine over maximum strength and force resistance [14]. For this, the subjects carried out a maximum repetition test (1MR test) in the bench press and squat exercises. They also carried out the maximum repetitions possible in the two exercises, with a relative load of 70% of the 1MR value. As in the previous study, the tests were conducted in four conditions, ingesting the supplementation 90 min before the tests, namely: 300 mg of caffeine (CAFF300); or 300 mg of theacrine (TEA300); or 150 mg of theacrine + 150 mg of caffeine (COMBO); or a placebo (PLA). The subjects’ fatigue and motivation levels were also evaluated. There was no difference between the groups. Only the CAFF300 group showed a significant increase in energy and motivation for the exercise.

Theacrine also does not seem to exert benefits in a chronic way, as demonstrated in a previous study. In this study, 8 weeks of pre-workout theacrine supplementation did not improve physical performance in strength, agility, sprint capacity, or aerobic and anaerobic performance [15].

To our knowledge, the present study was the first to acutely assess the effects of theacrine on agility, sprint capacity, and anaerobic performance in addition to aerobic and strength performance, measured in the studies of Bello et al. (2019) and Cesareo et al. (2019), respectively [13,14].

Although it is presented as a candidate for substituting caffeine, with the aim of promoting the same benefits as the latter, but without the side effects [23], our findings do not support the hypothesis of using theacrine for to improve physical performance. While there is solid evidence showing the benefits of caffeine in terms of increasing performance in different physical capacities in isolation, and also in specific physical actions in the sport environment [4], our findings corroborate the available literature in not justifying the use of theacrine for this purpose [13,14]. Even previous studies using higher dosages of the supplementation did not find any benefits of its use.

Some studies have shown that theacrine may be effective in improving focus or disposition in general, including disposition to exercise [9,11,24]. In addition to the benefits mentioned in clinical trials, animal model data have indicated that theacrine increases locomotive activity in rodents, without promoting tolerance of the body to supplementation, as occurs with caffeine [4,12]. The literature has also shown the safety of its use in humans [9]. In our study, the subjects reported no side effects. This study has limitations, such as the sample size, as well as the fact that the sample only consisted of male athletes. Furthermore, the study did not apply questionnaires or visual analogue scales. These questionnaires may be important to promote greater control over the subjects’ levels of fatigue and willingness to exercise. On the other hand, standardized training and feeding in our sample allowed for us to exert a great deal of control over this.

## 5. Conclusions

To our knowledge, this was the first study to assess the effects of theacrine on different physical capacities. Our findings corroborate the available literature in not finding any positive effects of theacrine supplementation on the subjects’ physical performance. However, we must consider the limitations of the study when considering our findings. It would be interesting for future studies to use larger samples; as well as for them to verify the effects of theacrine in larger doses, with different intake times before exercise, acutely as well as chronically.

## Figures and Tables

**Table 1:** General characteristics of the subjects.

Variable	Mean ± SD		
	Theacrine (*n* = 11)	Placebo (*n* = 11)	Total (*n* = 22)
Age (years)	19.64 ± 1.43	20.45 ± 2.11	20.05 ± 1.81
Height (cm)	178 ± 5.78	179 ± 8.5	178.27 ± 7.10
Body mass (kg)	76 ± 14.25	79.18 ± 16.37	78.57 ± 14.70
Lean mass (kg)	61.98 ± 9.89	63.58 ± 10.88	63.43 ± 9.96
Fat mass (%)	14.02 ± 4.97	15.60 ± 5.83	18.74 ± 3.88

cm = centimetres; kg = kilogrammes; % = percentage value.

**Table 2:** Performance of the subjects in the physical tests in the first and second assessments.

Variable	Theacrine (*n* = 11)			Placebo (*n* = 11)		
	Battery 1	Battery 2	Δ	Battery 1	Battery 2	Δ
Sextuple jump (m)	12.96 (±0.89)	13.09 * (±0.92)	0.13	12.18 (±1.31)	13.36 ** (±1.37)	0.18
Agility T test (s)	9.96 (±0.86)	9.91 (±0.85)	−0.05	9.94 (±0.73)	9.87 (±0.73)	−0.07
30 m sprint (s)	4.69 (±0.44)	4.66 * (±0.44)	−0.03	4.69 (±0.36)	4.65 ** (±0.35)	−0.04
40 s run test (m)	212.73 (±34.95)	215.36 * (±35.85)	2.63	225.45 (±22.07)	228.27 ** (±22.31)	2.82
Cooper test (m)	1745.45 (±391.21)	1761.09 * (±395.63)	15.64	1734.55 (±221.47)	1749.82 * (±223.51)	15.27

m = metres; s = seconds; * significant difference pre vs. post (*p* < 0.05); ** significant difference pre vs. post (*p* < 0.01).

## Data Availability

Data and publication materials are available from the corresponding author on reasonable request.
